# Case report: A rollercoaster journey of pemphigus vegetans

**DOI:** 10.3389/fimmu.2024.1481192

**Published:** 2025-01-13

**Authors:** Xiaoyuan Hou, Jia Chen

**Affiliations:** Department of Rheumatology, Shanghai Skin Disease Hospital, Tongji University School of Medicine, Shanghai, China

**Keywords:** immunobullous disease, pemphigus vegetans, IL-36 receptor antagonist, spesolimab, histopathology

## Abstract

Pemphigus vegetans (P Veg), the rarest subtype of pemphigus, is characterized by vegetative plaques, primarily affecting intertriginous areas. The most common autoantibodies target desmoglein 3 (Dsg3). A 60-year-old female patient presented with well-demarcated red vegetative plaques on her feet, vulva, and thigh, accompanied by surrounding pustules. Histopathological examination revealed epidermal hyperplasia with significant infiltration of neutrophils and eosinophils in the dermis. Enzyme-linked immunosorbent assay showed elevated anti-Dsg3 antibodies (203.2 U/ml), and immunohistochemical staining confirmed positive expression of anti-Dsg3 IgG antibodies in keratinocytes. The patient was diagnosed with P Veg and achieved remission after treatment with either 900 mg of intravenous spesolimab or oral methylprednisolone.

## Introduction

Pemphigus is an immunobullous disease characterized by the production of pathogenic autoantibodies that target the transmembrane glycoproteins of desmosomes, leading to acantholysis. Pemphigus vegetans (P Veg), the rarest subtype of pemphigus, accounts for only 1%–2% of all pemphigus cases (1). P Veg is characterized by vegetative plaques, primarily affecting the intertriginous areas (2). Clinically, P Veg is classified into two distinct subtypes: Hallopeau and Neumann. The former initially manifests as pustules, while the latter begins with vesicles (3). The most common autoantibodies in P Veg target desmoglein 3 (Dsg3), with some cases also involving Dsg1 (4). It is clinically essential to differentiate P Veg from conditions such as pyostomatitis pyodermatitis vegetans (PPV), IgA pemphigus, and other similar diseases. Here, we report the complicated diagnostic and therapeutic journey of a patient diagnosed with Hallopeau-type P Veg.

## Case description

**Figure 1:**
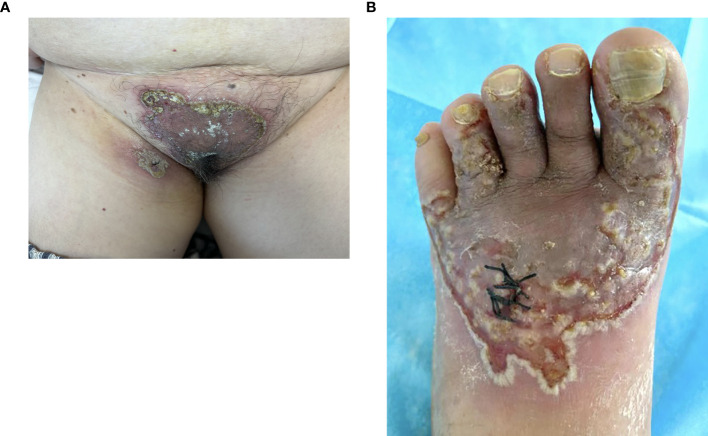
Vegetative plaques with circinate pustular edges on the vulva and thigh. The lesion at the biopsy site on the thigh showed significant exacerbation **(A)** and lesions on the toe, dorsum pedis **(B)**.

A 60-year-old woman presented to the clinic in January 2024 with reddish, well-defined, vegetative plaques surrounded by small pustules on her vulva and thigh. Notably, the lesions on her thigh worsened significantly after a biopsy (**Figure 1A**). Additionally, she had superficial erosions on her lower lip, but the oral mucosa itself was unaffected. The patient reported no significant family history of dermatological disorders, gastrointestinal symptoms, or inflammatory bowel disease (IBD). She also had no comorbidities, including hypertension or diabetes; no history of hematolymphoid disorders or other malignancies; and no record of long-term medication use. Vaccinations were up to date.

**Figure 2:**
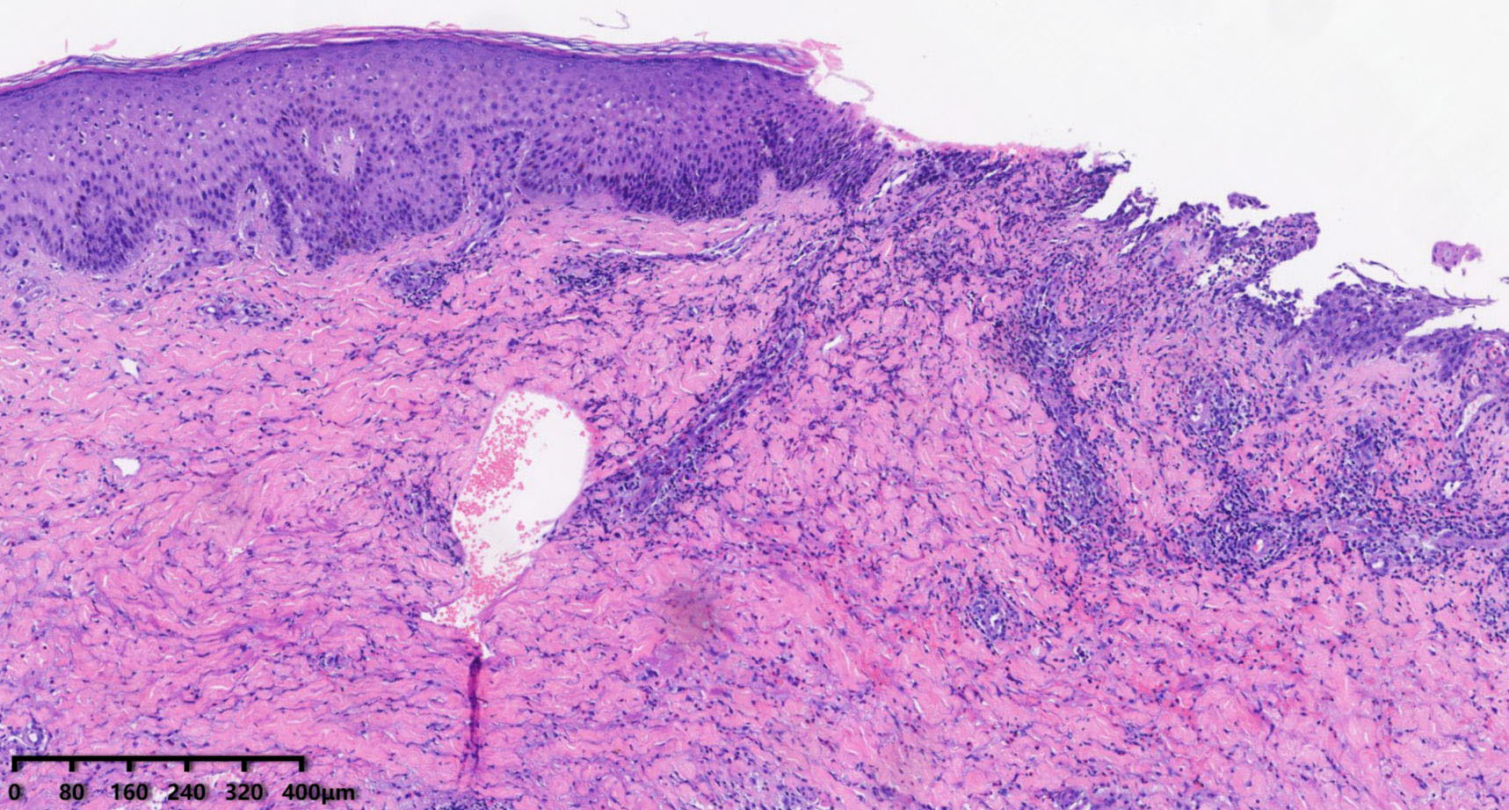
Skin biopsy (HE) showing mild epidermal hyperplasia, focal acantholysis, and dermal inflammation with neutrophils, lymphocytes, and eosinophils.

Upon further inquiry, the patient reported the onset of red plaques and pustular skin lesions on her toe and dorsum pedis approximately 10 months before her presentation (**Figure 1B**). Initial histopathological examination revealed mild epidermal hyperplasia and dermal inflammation with infiltration of neutrophils, lymphocytes, and eosinophils (**Figure 2**). Despite negative results for periodic acid–Schiff stain, acid–fast bacilli stain, and fungal cultures, the hospital initially suspected eczema complicated by a fungal infection. This suspicion was supported by the improvement of the skin lesions following empirical antifungal and antibacterial treatments. However, the rash continued to recur and persist. Due to multiple relapses, the patient underwent a second biopsy of the toe lesions, which revealed hyperkeratosis with parakeratosis, as well as inflammatory infiltration of lymphocytes, neutrophils, and eosinophils in the dermis. Based on these findings, acrodermatitis continua was suspected, and treatment with Leigongteng glucosides and thalidomide was initiated, leading to a favorable response. However, 1 month later, the patient developed the previously mentioned rash on the vulva and thigh.

## Diagnostic assessment

**Figure 3:**
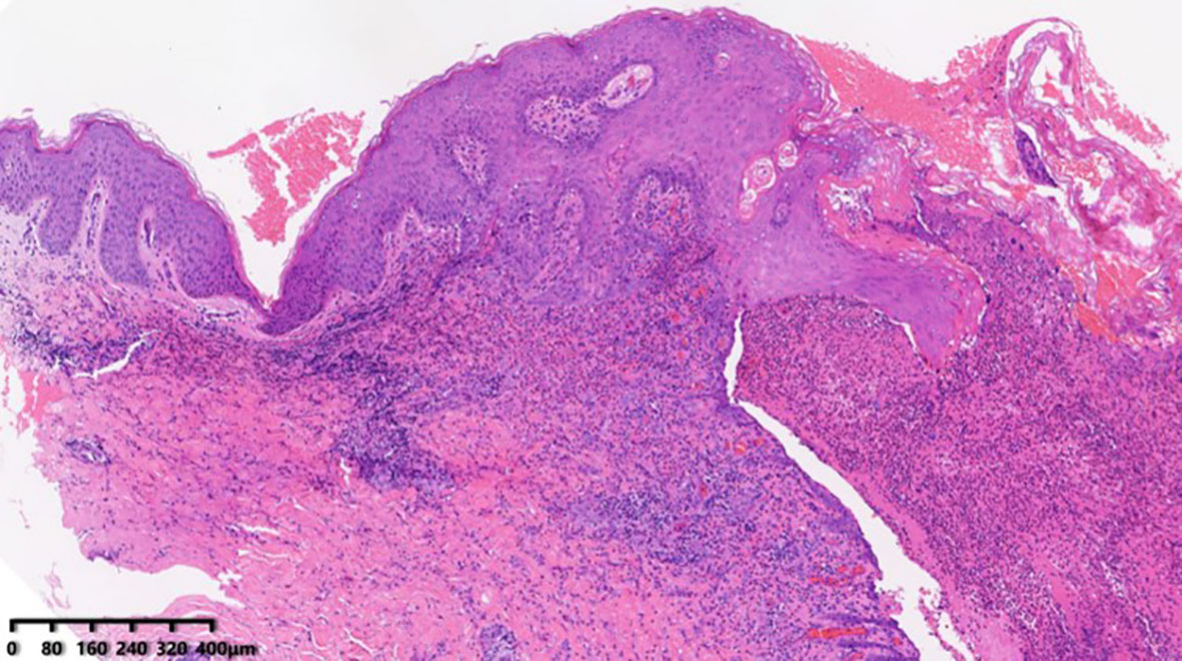
Skin biopsy (HE) showing epidermis erosions with extensive infiltrations of neutrophils, eosinophils, and lymphocytes in the superficial and mid-dermis.

During hospitalization, a differential blood count revealed elevated peripheral eosinophils (0.69 × 10^9/L) and an increased IgE level (208 IU/ml), with no other abnormalities. Histopathological examination of the thigh biopsy revealed epidermal hyperplasia, epidermis erosions, and extensive infiltration of neutrophils, eosinophils, and lymphocytes in the superficial and mid-dermis (**Figure 3**). Given the patient’s progressive disease course, the histopathological findings of suppurative inflammation, the exclusion of infections, tumors, and other relevant conditions, as well as the presence of localized pathergy, improvement with immunosuppressive therapy, and a PARACELSUS score of 11, we initially considered a diagnosis of pyoderma gangrenosum (PG) (5). We then administered spesolimab, an interleukin (IL)–36 receptor antagonist, at a dose of 900 mg. Following treatment, the lesions improved rapidly, with near-complete resolution within one week.

However, two months later, a flare-up of foot lesions occurred following treatment for an ingrown toenail, raising doubts about the accuracy of the initial diagnosis. A thorough review of the patient’s medical history and histopathological data revealed significant eosinophilic infiltration in all three biopsies. Additionally, focal acantholysis was observed in the first two samples. These findings were inconsistent with neutrophilic dermatoses such as acrodermatitis continua and PG. Based on these findings, we reevaluated the clinical presentation, which was characterized by intraepidermal pustules with eosinophilic infiltration and focal acantholysis. This led us to narrow the diagnosis to P Veg and PPV (6, 7). Subsequently, enzyme-linked immunosorbent assay revealed elevated anti-Dsg3 antibodies (203.2 U/ml), while anti-Dsg1 antibodies, anti-bullous pemphigoid (BP) 180 antibodies, and ant-BP 230 antibodies were undetectable (reference range <20). Immunohistochemical staining confirmed positive expression of anti-Dsg3 IgG antibodies in keratinocytes. Based on these findings, the diagnosis was ultimately revised to P Veg. The patient had a relatively small area of involvement. After receiving the recommended initial dose of prednisone/prednisolone (0.5 mg/kg daily), the patient was effectively managed with oral methylprednisolone at 24 mg daily, resulting in complete remission (8). The dose was then gradually reduced, and the patient is now maintained on 12 mg of oral methylprednisolone daily.

## Discussion

P Veg is the rarest variant of Pemphigus vulgaris, characterized by hypertrophic vegetative plaques and/or pustules. It primarily affects the intertriginous region, face, scalp, and rarely the nail beds (2). P Veg predominantly affects middle-aged adults. Depending on clinical features and disease courses, P Veg can be classified into two subtypes: Hallopeau type and Neumann type. The Hallopeau subtype is rare and typically follows a more indolent course, with vegetative plaques developing after pustules. In contrast, the Neumann subtype is more severe and refractory, initially presenting as vesiculobullous lesions resembling pemphigus vulgaris that later transform into vegetative plaques, often involving the oral mucosa (3, 9, 10). Elevated eosinophil levels are commonly observed in the serum of P Veg patients, and the predominant antibodies are anti-Dsg3 antibodies, which have a high specificity and sensitivity of 98%–100%. Dsg3 reactivity is nearly universally present in pemphigus patients but undetectable in unaffected individuals (11). Histologically, P Veg is characterized by hyperkeratosis, pseudoepitheliomatous hyperplasia, papillomatosis, acantholysis, and intraepithelial clefting. Neutrophilic and eosinophilic pustules are commonly found within the epidermis (12, 13). Direct immunofluorescence usually shows intercellular deposition of IgG and C3 (9).

In this case, the clinical presentation of vegetative plaques and pustules, combined with histopathological findings of epidermal hyperplasia, focal acantholysis, and eosinophilic infiltration, was accompanied by elevated levels of anti-Dsg3 antibodies in the serum. Immunohistochemical staining further confirmed the presence of anti-Dsg3 IgG antibodies in keratinocytes, collectively supporting the diagnosis of P Veg (13). Unfortunately, direct immunofluorescence was not performed due to the patient’s significant isomorphic responses (Köebner phenomenon), which caused marked deterioration of the skin lesions after each biopsy or other invasive procedure. However, we were able to confirm the positive expression of intercellular anti-Dsg3 IgG antibodies in keratinocytes through immunohistochemistry, which further validated the diagnosis of P Veg.

The differential diagnosis for pustules and vegetative plaques in intertriginous areas includes PPV and other forms of pemphigus, particularly IgA pemphigus. If mucosal involvement is present, paraneoplastic pemphigus should also be considered. PPV is a rare dermatosis that affects the skin and/or mucous membranes (7). Skin lesions typically present as exudative, erosive, or vegetative plaques with pustules and crusts. Mucosal involvement often affects the oral mucosa, presenting with erythema and multiple pustules that may rupture, forming shallow erosions and fissured ulcers, commonly referred to as “snail track.” PPV is strongly associated with IBD, especially ulcerative colitis (14, 15). Histologically, PPV is characterized by epidermal hyperplasia, focal acantholysis, and dense-mixed inflammatory infiltrates, with intraepidermal and subepidermal eosinophilic microabscesses. Direct immunofluorescence is typically negative (15, 16). Clinically and histologically, PPV closely resembles P Veg. However, in this case, the patient had no gastrointestinal symptoms, no personal or family history of IBD, and no significant oral mucosal involvement. Immunohistochemistry confirmed the deposition of anti-Dsg3 IgG antibodies in keratinocytes, and serum analysis revealed pemphigus antibodies, further supporting the diagnosis of P Veg. In this case, the differential diagnosis also included IgA pemphigus due to the pustular morphology. IgA pemphigus is a vesiculopustular disease in which skin lesions initially present as tense blisters, which later transform into pustules due to neutrophilic accumulation. Histopathologically, it is characterized by minimal or absent acantholysis and neutrophilic infiltration in the epidermis. Direct immunofluorescence typically shows intracellular IgA deposits within the epidermis (17, 18). In IgA pemphigus lesions, the infiltrating cells are predominantly neutrophils. However, this patient exhibited a predominance of eosinophilic infiltrates. Although direct immunofluorescence was not performed, immunohistochemistry confirmed the deposition of anti-Dsg3 IgG antibodies in keratinocytes, which further supports the exclusion of IgA pemphigus. Additionally, the patient had no history of hematolymphoid malignancies or other malignant disease, and there was no evidence of keratinocyte necrosis or interface dermatitis, further ruling out paraneoplastic pemphigus (19).

The diagnostic process for this patient was complex and challenging. Initially, the patient presented with localized plaques and pustules on the feet, and infection was suspected, but no pathogen was identified. Acrodermatitis continua and PG were then considered. Both conditions are neutrophilic dermatoses, but the abundant eosinophilic infiltration in the lesions was uncommon. Additionally, due to the diverse clinical manifestations of PG and the lack of definitive histopathological or laboratory findings, it remains a diagnosis of exclusion. The PARACELSUS score can aid in diagnosing PG, and although it initially suggested PG in this case, the diagnosis was ultimately proven to be incorrect. This is consistent with previous research, which shows that misdiagnosis or delayed diagnosis of PG is common (20). Therefore, patients suspected of having PG should undergo a thorough evaluation to exclude other potential diagnoses. Ultimately, after nearly a year-long journey, the diagnosis was revised to P Veg, primarily due to its rarity in clinical practice. The atypical onset on the feet and the significant isomorphic response further delayed the diagnosis. The absence of acantholysis in the histopathology also contributed to the delay in reaching a definitive diagnosis.

The first-line treatment for P Veg typically involves systemic corticosteroids and immunosuppressants (21), such as rituximab, mycophenolate mofetil, cyclosporine, azathioprine, methotrexate, cyclophosphamide or intravenous immunoglobulin (13). However, when the patient was diagnosed with PG and expressed concerns about long-term steroid use, we opted to initiate a trial of off-label spesolimab. This therapeutic agent, which specifically targets generalized pustular psoriasis, has also been approved for emergency investigational new drug use in PG (eIND 163533). Case reports have confirmed the effectiveness of spesolimab in treating PG (22), and in our case, it also showed good clinical efficacy. Interestingly, the diagnosis was eventually revised from PG to P Veg. Although no direct evidence currently links the IL-36 pathway to the pathogenesis of P Veg, studies have reported elevated IL-36α levels in various autoimmune blistering diseases, such as pemphigus vulgaris, dermatitis herpetiformis, and bullous pemphigoid, compared to healthy individuals (23). In this case, the efficacy of spesolimab may be attributed to increased IL-36 levels in autoimmune blistering diseases. Spesolimab binds with high affinity to the IL-36 receptor (IL-36R), blocking the IL-36 signaling pathway, reducing the release of inflammatory factors, inhibiting the aggregation and activation of inflammatory cells, and promoting the rapid resolution of pustules (24). However, further research is necessary to confirm IL-36 levels in P Veg patients and to evaluate spesolimab’s therapeutic potential.

Although the initial response to spesolimab was satisfactory, the abundant eosinophilic infiltration in the skin lesions and the limited clinical data on treating P Veg prompted us to reconsider our approach. Ultimately, we decided to administer systemic corticosteroid therapy, considering its proven efficacy and cost-effectiveness. After receiving the recommended initial dose of prednisone/prednisolone (0.5 mg/kg daily), the patient’s condition was effectively managed, leading to complete remission.

Early recognition and accurate diagnosis of this rare subtype of pemphigus are essential for enabling targeted therapy and achieving early clinical remission, with histopathology playing a crucial role. Key diagnostic clues, such as abundant eosinophilic infiltration and focal acantholysis, should be carefully considered. Last, the efficacy of spesolimab in P Veg requires further validation through additional clinical data, and its underlying mechanisms warrant further exploration.

## Data Availability

The raw data supporting the conclusions of this article will be made available by the authors, without undue reservation.
